# Reduction of Long‐Term Care Dependence After an 8‐Year Primary Care Prevention Program for Stroke and Dementia: The INVADE Trial

**DOI:** 10.1161/JAHA.112.000786

**Published:** 2012-08-24

**Authors:** Horst Bickel, Karl‐Heinz Ander, Monika Brönner, Thorleif Etgen, Hans Gnahn, Othmar Gotzler, Holger Poppert, Klaus Pürner, Dirk Sander, Hans Förstl

**Affiliations:** From the Department of Psychiatry, Technische Universität München, Klinikum rechts der Isar, Munich, Germany (H.B., M.B., H.F.); From the Department of Neurology, Technische Universität München, Klinikum rechts der Isar, Munich, Germany (H.P.); the INVADE Study Group, Baldham, Germany (K.-H.A., H.G., O.G., K.P.); the Department of Neurology, Klinikum Traunstein, Traunstein, Germany (T.E.); the Department of Neurology, Benedictus Krankenhaus Tutzing, Tutzing, Germany (D.S.)

**Keywords:** stroke, prevention, risk factors, dementia

## Abstract

**Background:**

Stroke and dementia are the major causes for long‐term care (LTC) dependence in old age. This intervention trial compared a multidomain prevention program for stroke and dementia with usual medical care in reducing the need for LTC.

**Methods and Results:**

The Intervention Project on Cerebrovascular Disease and Dementia in the District of Ebersberg (INVADE) was a general practice–based 8‐year trial in 2 defined catchment areas in Upper Bavaria, Germany. All 11 317 insurants of a statutory health insurance plan who were ≥55 years of age and lived in the intervention district were offered the opportunity to participate in a prevention program; 3908 enrolled. The 13 301 insurants in the reference district received usual medical care. The intervention consisted of the systematic identification and evidence‐based treatment of vascular risk factors. The primary clinical end point was incidence of LTC dependence according to external assessment by a special medical service in the framework of the statutory German LTC insurance. Age‐ and sex‐specific incidence rates from the reference district were used to calculate the expected number of cases of LTC dependence under usual medical care. The expected number was compared with the observed number of cases in the intervention district. Analysis was by intention to treat. During the 5 years after completion of the recruitment period, significantly fewer incident cases of LTC dependence arose in the intervention district than expected (χ^2^=13.25; *P*<0.001). In women, the incidence was reduced by 10% (*P*<0.01). In men, the incidence was reduced by 9.6% (*P*<0.05).

**Conclusions:**

Our results support the feasibility and effectiveness of a primary care prevention program for stroke and dementia to reduce the risk of developing LTC dependence.

**Clinical Trial Registration:**

URL: http://www.clinicaltrials.gov. Unique identifier: NCT01107548. **(*J Am Heart Assoc*. 2012;1:e000786 doi: 10.1161/JAHA.112.000786.)**

## Introduction

troke and dementia are the leading causes of lasting disability, need for long‐term care (LTC), and reliance on nursing homes.^1–4^ Studies of illness costs show them to be the most expensive diseases of advanced age.^5,6^ Because the incidence rates of stroke and dementia increase rapidly with advancing age,^7–9^ a large increase in the number of patients must be expected as a result of the continuous aging of the population unless the risk of these diseases can be reduced by preventive measures.

The INTERSTROKE study found that 90% of stroke risk is determined by 10 risk factors.^10^ Among the most important modifiable risk factors for stroke, with a high population‐attributable risk and a proven preventive effect of their treatment, are arterial hypertension, smoking, diabetes mellitus, atrial fibrillation, and dyslipidemia.^11^ Other factors, such as obesity, lack of physical activity, alcohol abuse, hyperhomocysteinemia, and depression, are independently associated with the risk of stroke, but there is still a lack of controlled studies that can unequivocally demonstrate that modification of these factors has a preventive effect.^11,12^

Observational studies from the past 2 decades report connections of dementia with virtually all risk factors that also increase the risk of stroke. Not only the vascular dementias but also the more frequent Alzheimer's dementia are closely associated with cardiovascular risk factors.^13,18^ Future dementia can be predicted most accurately by vascular risk factors,^19,20^ and persons who have a higher stroke risk according to a validated stroke risk scale exhibit more serious cognitive deficits.^21,22^ Thus, the evidence for a causal role of cardiovascular risk factors in dementia is strong, but the examination of various preventive strategies has yielded inconsistent results.^23,25^ Against the background of a multifactorial etiology of dementia, experts in the field have been calling for multidomain interventions and a lowering of the vascular risk profile by evidence‐based treatment.^15,17,23,26^

The aim of the present study was to investigate whether the systematic identification and treatment of vascular risk factors, which are amenable to intervention in the primary care setting, can reduce the occurrence of the need for LTC. We chose LTC dependence as the primary clinical end point because this parameter is readily available in Germany as a result of statutory LTC insurance. Under the assumption that stroke and dementia together cause most of the cases of LTC dependence in the population, we hypothesized that an effective prevention program against stroke and dementia would be connected with a reduction of the need for LTC.

## Methods

### Study Population and Participants

**Figure 1.**
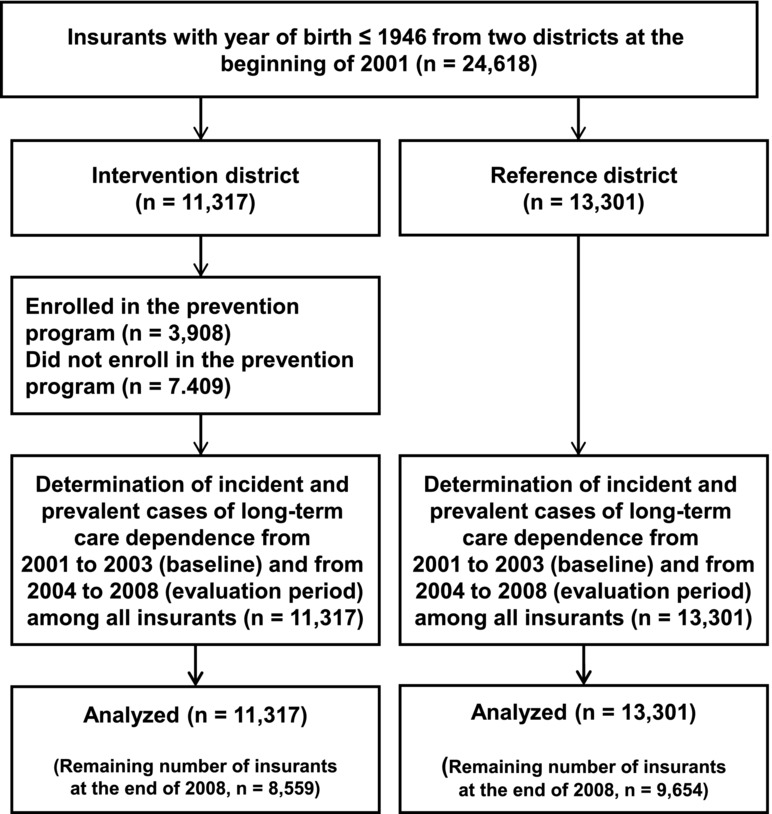
Composition of the study population.

The Intervention Project on Cerebrovascular Disease and Dementia in the District of Ebersberg (INVADE) is a prevention study at the level of medical primary care in a geographically defined catchment area in Upper Bavaria, Germany. All members of the AOK Bayern (Allgemeine Ortskrankenkasse Bayern) health insurance company who were ≥55 years of age (year of birth ≤1946) and lived in the district of Ebersberg were eligible for the study. The AOK is the largest health insurance company in Germany, with a market share >40% in Bavaria. At the beginning of the study, the district, which is located to the east of Munich, had a total population of nearly 119 000, out of which 11 317 of the insured fulfilled the inclusion criteria at the beginning of the year 2001 (Figure 1).

**Figure 2.**
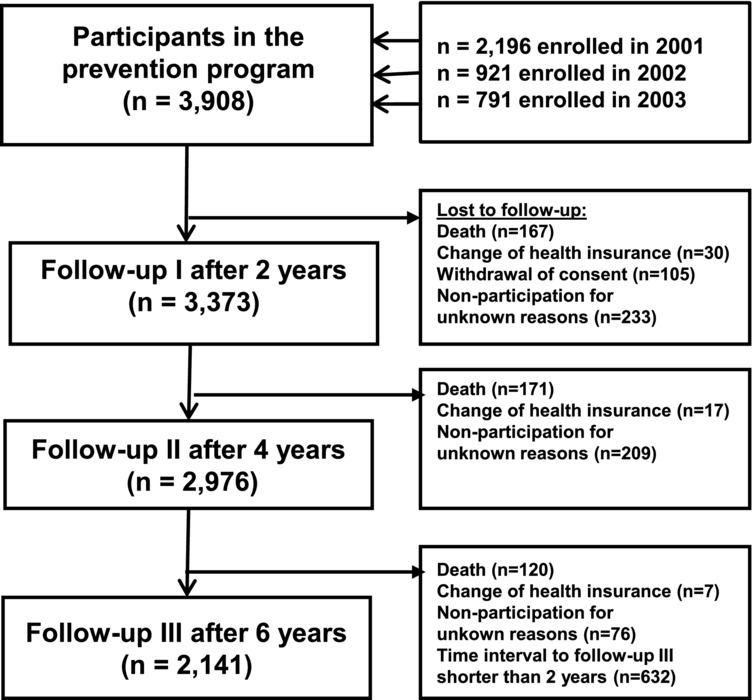
Flow of participants in the intervention program.

After they had been invited to participate in writing by their health insurer, a total of 3908 people enrolled in the trial during the recruitment phase, which took place between 2001 and 2003 (Figure 2). The remaining 7409 did not enroll in the trial. In reference to the number of insurants at the outset, a total of 34.5% participated: 33.2% of the men and 35.5% of the women. Among those 55 to 69 years of age, the participation rate was 40.3%; among those 70 to 79 years of age, it was 34.4%; and among those ≥80 years of age, it was 15.9% (Table 1).

**Table 1.** Number of Participants in the Intervention Program and Number of Insured Persons in the Intervention District at the Beginning of 2001 by Age Group and Sex

Year of Birth	Participants		Insured Persons	
		
	Men, n	Women, n	Men, n	Women, n
1942–1946	286	357	933	813
1937–1941	487	607	1288	1261
1932–1936	371	444	998	1043
1927–1931	245	388	701	982
1922–1926	129	285	447	913
1917–1921	57	152	252	698
1912–1916	20	63	133	472
≤1911	5	12	72	311
≤1946	1600	2308	4824	6493

### Reference Population

A cluster randomization of the general practitioner (GP) practices in the study area was not feasible because the insured have the free choice of their doctors in the German healthcare system. In view of the intended study duration of 8 years, a high level of transfer of the insured among the various practices was to be expected, and the offer of an intervention might possibly have increased such patient movement. This would have prevented a comparison of participants and nonparticipants in the intervention program. We therefore decided to compare the intervention district with a second, structurally similar district in Upper Bavaria in which the intervention program was not offered.

As a reference district with usual medical care, the district of Dachau was chosen. At the beginning of 2001, Dachau had a population of 129 500, with 13 301 AOK‐insured patients who were born in 1946 or earlier (Table 2). The 2 districts have no common borders, and they are separated by the city of Munich, so it would be unlikely that a patient from one district would receive medical care in the other district. Both districts are rural in character and very similar with regard to area, population, age and sex structure, migration rates, and number of places in nursing homes. With the use of the health insurance company's data, morbidity and mortality rates were compared between the districts before the beginning of the study. For the year 2000, which preceded the study, morbidity and mortality rates among those insured by the AOK were not significantly different. In the intervention district, 959 prevalent cases of LTC dependence were registered at the end of the year; during the course of the year, 273 incident cases of LTC dependence and 394 deaths had occurred. After age‐ and sex‐specific standardization of the rates of the reference district, 1012 prevalent (χ^2^=2.78, *P*=0.10) and 273 incident cases of LTC dependence (χ^2^=0.0, *P*=0.99) as well as 426 deaths (χ^2^=2.40, *P*=0.12) would have been expected. In the 3 subsequent years, 2001 through 2003, during which the study participants were recruited, the comparability of the 2 districts was confirmed. During this baseline phase, no significant differences in death rate or in the incidence and prevalence of LTC dependence occurred (see Results section).

**Table 2.** Number of Insured Persons With Birth Year ≤1946 at the Beginning of the Year 2001 in the 2 Districts, by Age Group and Sex

Year of Birth	Intervention District		Reference District	
		
	Men, n	Women, n	Men, n	Women, n
1942–1946	933	813	1018	1180
1937–1941	1288	1261	1434	1428
1932–1936	998	1043	1054	1275
1927–1931	701	982	829	1151
1922–1926	447	913	526	1120
1917–1921	252	698	277	775
1912–1916	133	472	170	622
≤1911	72	311	80	362
≤1946	4824	6493	5388	7913

### Assessment of Vascular Risk Factors

Data were obtained at baseline through participants’ self‐report, examination by the family doctor, laboratory analyses, and duplex ultrasonography (Table 3). Of the total of 76 GPs and family doctors (designated together as *GPs* hereafter) who practiced in the district of Ebersberg, 72 (94.7%) participated in the study. The investigation by the GP included a standardized questionnaire, a physical examination, a 12‐lead electrocardiogram, and an overnight fasting venous blood sample for analysis in a central laboratory. Cognitive deficits were assessed through the 6‐Item Cognitive Impairment Test (6CIT), a short cognitive screening test designed especially for use in general practice.^28,29^ Restrictions of activities of daily living were estimated according to the Rankin Scale^30^ and the Barthel Index.^31^ Fasting blood samples were transferred on ice to a central laboratory that performed all analyses.

**Table 3.** Data Sources and Study Variables

Data Source	Study Variables
Participants’ self‐report	Sociodemographic data
	Subjective health
	Use of medical services
	Memory complaints
	Depressive symptoms (Geriatric Depression Scale)^27^
GP	History of diseases
	Current diseases
	Medication
	Smoking status
	Alcohol consumption
	Physical activity
	Body mass index ([weight in kilograms] / [height in meters^2^])
	6‐Item Cognitive Impairment Test (6CIT)^28^
	Impairment in activities of daily living (Rankin Scale, Barthel Index)^31^
	Blood pressure
	Ankle‐to‐brachial index
	Electrocardiogram
Fasting blood sample	Total cholesterol
	Low‐density lipoprotein cholesterol
	High‐density lipoprotein cholesterol
	Triglycerides
	Serum glucose
	Hemoglobin A_1c_
	Creatinine
	Homocysteine
	High‐sensitivity C‐reactive protein
Ultrasound imaging	Intima‐media thickness of common carotid artery^2000^

Duplex ultrasonography of the carotid arteries was performed by 8 certified investigators. Mean common carotid artery intima‐media thickness was measured as previously described^32^ with the use of a computer‐supported image analysis system (Sigmascan Pro 5.0).

Every 3 months after baseline, the GP measured the blood pressure of each participant, documented the current drug therapy, and reported whether vascular events had occurred in the preceding months. At intervals of 2, 4, and 6 years after baseline, a comprehensive follow‐up examination was scheduled, in which a complete measurement of the risk factors was to be performed.

### Intervention

The intervention consisted of the repeated, systematic identification of vascular risk factors and their subsequent treatment by the GP according to evidence‐based guidelines. For each participant, an individual risk profile that included the Framingham Stroke Risk Profile^33^ was reported to the GP, together with treatment goals and recommendations based on the current guidelines of the medical societies. A compact summary of the treatment recommendations was made available to the GPs in the form of a guideline brochure developed specially for this purpose; the brochure described the threshold values, target values, and therapy recommendations for arterial hypertension, dyslipidemia, and diabetes mellitus, as well as the therapy options for atrial fibrillation and depression. The GPs were trained in the use of the guidelines and were to orient the treatment of the participants according to these guidelines. The GPs also worked toward a change of the participants’ lifestyles, with the aims of smoking cessation, reduction of risky alcohol consumption, increase in physical activity, reduction of obesity, and improvement in nutrition according to the example of Mediterranean diet.^34^

The study protocol was approved by the ethics committee of the medical faculty at the Technische Universität München. Written informed consent was obtained from each participant after complete information about the study had been provided.

### Outcome Measures

The primary clinical end point chosen in the present study was the incidence of LTC dependence. In the year 1995, Germany introduced a statutory LTC insurance. Not only the elderly but also people of all age groups in need of care are eligible for benefits from the LTC insurance. These benefits are not coupled to any specific form of care but are furnished when care is given by relatives or other private caregivers as well as when care is given by professional ambulatory care services or by resident care in nursing homes.

To receive benefits from the LTC insurance, a persistent dependence on the assistance of caregivers must be proved. The health insurance fund's medical service departments examine the applicants in their homes and assess the extent of their need for care in the areas of personal hygiene, eating, mobility, and housekeeping, according to legally defined uniform criteria.^35,36^ Information on the number of patients with LTC dependence was made available to us by the LTC fund as aggregated data itemized according to sex and year of birth.

The secondary clinical end points used were the temporal changes in blood pressure, fasting serum glucose, hemoglobin A_1c_, total cholesterol, low‐density lipoprotein cholesterol, high‐density lipoprotein cholesterol, triglycerides, homocysteine, and body mass index after 2 and 4 years in the group of participants. Changes over 6 years were not analyzed in detail because for many participants the third follow‐up examination laid beyond the legal maximum permissible study duration of 8 years for model projects of the health insurers.

### Sample Size Calculation

We offered all policyholders in the district of Ebersberg the opportunity to participate in the program (“intention to treat”), although it was clear that only a certain percentage would enroll. We therefore attempted to estimate the least number of patients that would have to participate for a significant difference between all insured in the 2 districts to be observable. Under usual treatment, we expected ≍280 new cases of LTC dependence per calendar year in the district of Ebersberg. For the 5‐year evaluation phase after recruitment of the participants, there thus would have resulted a total number of 1400 incident cases. With a level of significance of *P*<0.05 and a statistical power of 80%, the expected number of 1400 incident cases would have to be reduced by ≥88. Under the assumption that the intervention would lower the risk by 25%, the participation of ≥2845 insurants in the intervention program was required for this reduction of 88 cases. Because it is difficult to judge beforehand whether the participants are at the same risk as the total population of insurants, we aimed for a participant number of substantially more than 3000.

### Statistical Analyses

Statistical analyses were performed with SPSS for Windows, version 18.0. Data are described as means and standard deviations (mean±SD) or as numbers and percentages. For the analysis of secondary end points, we used repeated‐measures analysis of variance. The χ^2^ test was used for the analysis of the primary end point (incidence of LTC dependence) on the basis of the aggregated data. LTC dependence comprises both those persons cared for in private homes and those accommodated in nursing homes.

On the basis of year of birth (1942–1946, 1937–1941, 1932–1936, 1927–1931, 1922–1926, 1917–1921, and ≤1916) and sex, we formed 14 subgroups in each district. For the reference district, we calculated the incidence rates of LTC dependence under usual medical care. In each subgroup, we divided the incident cases of LTC dependence that developed in the respective calendar year (numerator) by the number of insurants at the beginning of the year less the prevalent cases (denominator). We multiplied the resulting age‐ and sex‐specific incidence rates by the number of insurants at risk in the intervention district and summed the products. Using this standardization, we obtained for each calendar year the number of incident cases that were to be expected if no intervention had taken place. This expected number of incident cases in the years 2004–2008 was compared to the number of new cases that in fact occurred in the intervention district. A *P* value of 0.05 was considered significant. To determine whether the risk of LTC dependence already differed between the districts during the recruitment period, we performed the same comparison for the years 2001–2003.

Analysis was by intention to treat. It is important to note that the expected number of LTC dependence cases was not calculated for the subsample of the study participants but rather for the totality of the insured in the intervention district, regardless of whether they had participated in the intervention program. That is, the incident cases of LTC dependence among all 11 317 insured in the district of Ebersberg were compared with the expected number, which was calculated according to the standardized rate from the reference district. The comparisons were carried out for both sexes together and separately for men and women. In addition, we calculated the standardized morbidity ratio as the quotient of the observed and expected incidence and its 95% confidence interval.

Furthermore, we compared the expected and the observed numbers of prevalent cases of LTC dependence for each individual calendar year. Prevalence rates in the reference district were calculated as the number of prevalent cases at the end of the year in the 14 subgroups formed according to age and sex (numerator) divided by the number of insured in these subgroups at the end of the year. The expected number of prevalent cases was determined, as in the case of incidence, through multiplication of the age‐ and sex‐specific rates in the reference district by the number of insured in the intervention district.

Death represents a competing risk and was likewise compared for the entire period of the study. In the reference district, age‐ and sex‐specific death rates were calculated for each calendar year by dividing the number of deaths in the course of each year (numerator) by the number of insured at the beginning of the year (denominator). The expected number of deaths in the intervention district resulted from transfer of the rates to the age and sex structure of the insured in the district of Ebersberg. This expected number was compared with the observed number of deaths.

## Results

### Baseline Data

The mean age of the 3908 participants in the intervention program was 67.7 years (standard deviation, 7.8); their ages ranged from 55 to 102 years. Half of the sample had lived for >50 years in the district of Ebersberg and 94% for ≥10 years. Only 2.8% had not consulted the GP at all in the past year, and 19.5% consulted the GP less than once per quarter‐year (Table 4).

**Table 4.** Baseline Characteristics of Participants

Characteristics	Men* (n=1600)	Women* (n=2308)	Total* (n=3908)
Age, mean±SD, y	66.7±7.1	68.5±8.1	67.7±7.8
Duration of residency in district, mean±SD, y	44.4±20.0	45.5±20.7	45.0±20.1
GP consultation less than once a quarter	391/1595 (24.5)	476/2295 (20.7)	867/3890 (22.3)
Inpatient treatment in past 12 months	371/1597 (23.2)	506/2301 (22.0)	877/3898 (22.5)
Subjective health (fair or poor)	603/1598 (37.7)	849/2302 (36.9)	1452/3900 (37.2)
Depression (Geriatric Depression Scale >5)	146/1588 (9.2)	233/2273 (10.3)	379/3861 (9.8)
Cognitive impairment (6CIT >7)	191/1599 (11.9)	227/2307 (9.8)	418/3906 (10.7)
Current smoking	259/1598 (16.2)	138/2307 (6.0)	397/3905 (10.2)
No or little physical activity	808/1597 (50.6)	1301/2307 (56.4)	2109/3904 (54.0)
Obesity (body mass index >30.0 kg/m^2^)	391/1600 (24.4)	616/2307 (26.7)	1007/3907 (25.8)
Systolic blood pressure			
≥140 mm Hg	795/1600 (49.7)	1172/2308 (50.8)	1967/3908 (50.3)
≥160 mm Hg	242/1600 (15.1)	362/2308 (15.7)	604/3908 (15.5)
Diastolic blood pressure			
≥90 mm Hg	405/1600 (25.3)	542/2308 (23.5)	947/3908 (24.2)
≥100 mm Hg	101/1600 (6.3)	120/2308 (5.2)	221/3908 (5.7)
Total cholesterol ≥6.21 mmol/L	353/1591 (22.2)	716/2283 (31.8)	1081/3874 (27.9)
Low‐density lipoprotein cholesterol ≥4.14 mmol/L	275/1547 (17.8)	501/2259 (22.2)	776/3806 (20.4)
Triglycerides ≥2.26 mmol/L	370/1589 (23.3)	294/2283 (12.9)	666/3872 (17.2)
Fasting serum glucose ≥7 mmol/L	185/1590 (11.6)	200/2284 (8.8)	385/3874 (9.9)
Hemoglobin A_1c_ >6.5%	189/1592 (11.9)	251/2279 (11.0)	440/3871 (11.4)
C‐reactive protein >28.5 nmol/L	544/1590 (34.2)	870/2284 (38.1)	1414/3874 (36.5)
Intima‐media thickness of the common carotid artery ≥1 mm	241/1385 (17.4)	209/2001 (10.4)	450/3386 (13.3)
Ankle‐to‐brachial‐index <0.9	240/1522 (15.8)	374/2228 (16.8)	614/3750 (16.4)
Homocysteine >12 μmol/L	92/1457 (6.3)	156/2142 (7.3)	248/3599 (6.9)
Diseases as reported by GP			
Dementia	32/1589 (2.0)	45/2301 (2.0)	77/3890 (2.0)
Stroke	67/1596 (4.2)	68/2301 (3.0)	135/3897 (3.5)
Chronic kidney disease	69/1590 (4.3)	53/2297 (2.3)	122/3887 (3.1)
Alcohol abuse	109/1583 (6.9)	16/2303 (0.7)	125/3886 (3.2)
Myocardial infarction	115/1592 (7.2)	47/2300 (2.0)	162/3892 (4.2)
Transient ischemic attack	79/1587 (5.0)	95/2299 (4.1)	174/3886 (4.5)
Atrial fibrillation	94/1590 (5.9)	91/2305 (3.9)	185/3895 (4.7)
Peripheral vascular occlusion	131/1564 (8.4)	52/2269 (2.3)	183/3833 (4.8)
Coronary heart disease	264/1553 (17.0)	216/2258 (9.6)	480/3811 (12.6)
Depression	136/1594 (8.5)	366/2292 (16.0)	502/3886 (12.9)
Diabetes mellitus	373/1593 (23.4)	416/2305 (18.0)	789/3898 (20.2)
Dyslipidemia	691/1578 (43.2)	1003/2277 (44.0)	1694/3855 (43.9)
Hypertension	883/1593 (55.4)	1359/2301 (59.1)	2242/3894 (57.6)

Data are expressed as number of participants / sample size (percentage) unless otherwise indicated.

* Because of missing values, sample sizes may differ.

Arterial hypertension (57.6%), dyslipidemia (43.9%), and diabetes mellitus (20.2%) were reported frequently by the GP.

Many cases of disease were, however, unknown or insufficiently treated. Only 32.5% of the patients with known hypertension had baseline blood pressure in the target range of <140/90 mm Hg; in 41.9% of the cases, values up to 159/99 mm Hg were measured, and in 25.6%, blood pressure values exceeded 160/100 mm Hg. Furthermore, high blood pressure that previously had been unknown and untreated was diagnosed at baseline for 588 (15.0%) participants. For 393 (10.1%) participants, dyslipidemia was discovered for the first time, and for 65 (1.7%), previously undiscovered diabetes mellitus was identified.

### Temporal Changes in Risk Factors

Among the participants in the intervention program, blood pressure, serum glucose, total cholesterol, low‐density lipoprotein cholesterol, and triglycerides decreased, particularly during the first 2 years of the intervention, whereas high‐density lipoprotein cholesterol increased. Hemoglobin A_1c_, homocysteine, and body mass index decreased only in the first 2 years after baseline and then increased again (Table 5). The changes were accompanied by increased drug use. The use of antihypertensive medications went up, from 57.4% at baseline to 70.6% at the second follow‐up; the use of statins increased from 16.1% to 25.9%, and the use of anticoagulant and antiplatelet drugs rose from 31.0% to 43.5%.

**Table 5.** Changes in Risk Factors Between Baseline and First and Second Follow‐Up Among Participants in the Intervention Program (n=2906 With Complete Data)

Risk Factor	Baseline (mean±SD)	Follow‐Up I (mean±SD)	Follow‐Up II (mean±SD)	*F* Value*† (*df*)	*P*
Systolic blood pressure, mm Hg	139.4±17.8	137.1±17.4	136.9±17.1	31.2 (2, 2904)	<0.001
Diastolic blood pressure, mm Hg	82.2±9.4	80.7±8.9	80.0±9.2	57.0 (2, 2904)	<0.001
Fasting serum glucose, mmol/L	5.30±1.65	5.07±1.51	5.05±1.64	47.1 (2, 2845)	<0.001
Hemoglobin A_1c_, %	5.83±0.86	5.73±0.71	5.98±0.70	431.2 (2, 2826)	<0.001
Total cholesterol, mmol/L	5.67±1.03	5.60±1.00	5.53±1.03	27.3 (2, 2844)	<0.001
Low‐density lipoprotein cholesterol, mmol/L	3.44±0.90	3.32±0.87	3.25±0.89	62.2 (2, 2728)	<0.001
High‐density lipoprotein cholesterol, mmol/L	1.51±0.41	1.56±0.41	1.59±0.42	119.1 (2, 2839)	<0.001
Triglycerides, mmol/L	1.62±0.96	1.60±0.94	1.57±0.88	13.7 (2, 2842)	<0.001
Homocysteine, μmol/L	7.36±3.32	6.47±3.71	6.62±4.40	67.6 (2, 2605)	<0.001
Body mass index, kg/m^2^	27.84±4.39	27.73±4.47	27.74±4.61	6.64 (2, 2898)	0.002

* Repeated‐measures analysis of variance.

† Degrees of freedom may differ because of missing data.

### Effects on LTC Dependence

**Figure 3.**
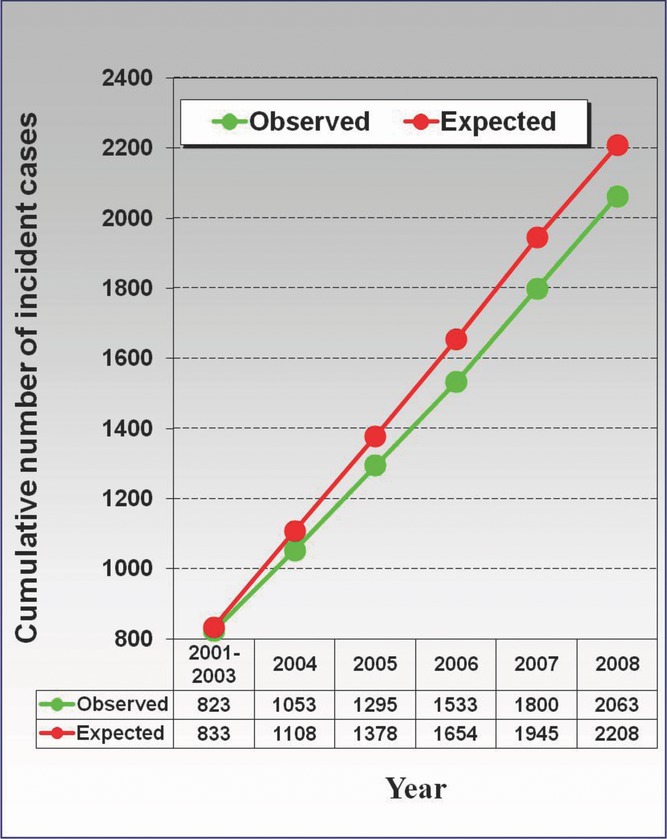
Cumulative numbers of observed and expected incident cases of LTC dependence during the years 2001 to 2008 in the intervention district (n=11 317 at the beginning of 2001).

Comparisons of incidence and prevalence of LTC dependence refer to the entire populations in the 2 districts. In the years 2004–2008, a total of 1240 incident cases of LTC dependence were observed in the intervention district. According to the data from the reference district, 1375 new cases were to be expected (Figure 3). The difference of 135 cases (−9.8%) is highly significant (χ^2^=13.25, *df*=1, *P*<0.001). The reduction of the incident cases of LTC dependence was significant for both men and women. Among the men, 439 new cases were observed, whereas without intervention, 485 were to be expected (χ^2^=4.36, *df*=1, *P*=0.037). Among the women, 801 new cases were observed, whereas 890 were to be expected (χ^2^=8.90, *df*=1, *P*=0.003). In the total population, the standardized morbidity ratio amounted to 0.90 (95% confidence interval, 0.85–0.95). Among the men, the standardized morbidity ratio was 0.905 (95% confidence interval, 0.82–0.99), and among the women, the standardized morbidity ratio was 0.90 (95% confidence interval, 0.84–0.96). During the baseline phase (2001–2003), no significant difference occurred (χ^2^=0.12, *df*=1, *P*=0.73).

**Figure 4.**
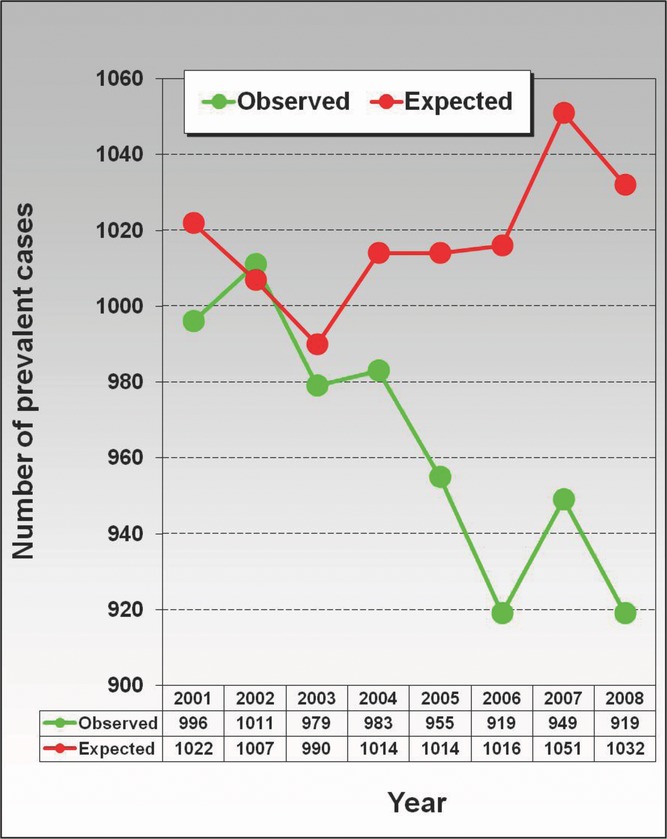
Numbers of observed and expected prevalent cases of LTC dependence at the end of each of the years 2001 to 2008 in the intervention district (n=11 317 at the beginning of 2001).

The number of prevalent cases of LTC dependence was lower than would have been expected without intervention (Figure 4). The difference was not significant at the end of the years 2004 (χ^2^=0.95, *df*=1, *P*=0.33) and 2005 (χ^2^=3.43, *df*=1, *P*=0.06). However, in the years 2006 (χ^2^=9.26, *df*=1, *P*=0.002), 2007 (χ^2^=9.90, *df*=1, *P*=0.002), and 2008 (χ^2^=12.37, *df*=1, *P*<0.001), the prevalence was significantly lower than expected. On average, there were 80 (−7.8%) fewer prevalent cases than expected between 2004 and 2008 in the intervention district. During the recruitment phase, comprising the years 2001 (χ^2^=0.66, *df*=1, *P*=0.42), 2002 (χ^2^=0.02, *df*=1, *P*=0.89), and 2003 (χ^2^=0.12, *df*=1, *P*=0.73), we found no significant differences between the 2 districts.

During the baseline phase (2001–2003), there were no significant differences in death rate. In the intervention district, 1216 deaths occurred, whereas 1270 would have been expected (χ^2^=2.30, *df*=1, *P*=0.13). In the evaluation phase (2004–2008), on the other hand, the death rate in the intervention district was significantly reduced. In this period, only 1939 deaths occurred, whereas 2112 would have been expected (χ^2^=14.17, *df*=1, *P*<0.001).

## Discussion

To the best of our knowledge, INVADE is the first trial on the prevention of stroke and dementia in primary care that has demonstrated an effect of a tailored multidomain intervention on the incidence of LTC dependence. The results show that significantly fewer new cases of LTC dependence occurred among insurants of a statutory health insurance company who were ≥55 years of age and received an offer to participate in a GP‐driven prevention program for the reduction of cardiovascular risk factors than occurred among a reference group of insurants who received usual treatment and care. During the 5‐year evaluation period, there were 9.8% fewer new cases of LTC dependence among the insurants who lived in the intervention district than were to be expected according to the incidence rates in the reference group. The risk reduction was equally strong for women and men. This favorable outcome also was reflected in the comparison of the prevalent cases of LTC dependence. From the third year after baseline, the prevalence for the remaining study duration was significantly reduced, by 9.5% in the third year, 9.7% in the fourth year, and 10.9% in the fifth year of the evaluation period.

This strong effect was found for the comparison of the total population of insurants ≥55 years of age in the intervention and reference districts, even though only 34.5% of the insurants who were invited to participate in the prevention program actually enrolled. This could be explained in one of 2 ways: (1) The effect among participants in the prevention was substantially stronger to bring about the present differences in the total population, or (2) the nonparticipants profited from trial‐driven improvements in the management of vascular risk factors by the GP.

A significant preventive effect was achieved even though a large majority of the participants already had been in frequent contact with their GPs before the beginning of the trial. This could suggest that the diagnosis and treatment of medical risk factors in routine primary care are not always carried out with optimum diligence. This was further underlined by the discovery of a considerable number of risk factors that previously had been unknown and untreated. This phenomenon stresses the preventive potential of systematic programs.^37–39^

It remains unclear whether the risk of dementia can be reduced by the intervention or whether the reduction in LTC dependence was caused by a reduction of the risk of vascular diseases or by completely different mechanisms. For stroke, an effect of primary prevention measures is supported by evidence from randomized trials.^2002^ Because a transition from stroke to dementia is not rare,^41,42^ a reduced risk of poststroke dementia is probable. It is, however, unknown whether the occurrence of degenerative dementias unrelated to the consequences of stroke can be prevented. Today, controlled trials do not yield sufficient evidence to develop effective intervention strategies against dementia at the population level.^2008^ Reviews found no convincing evidence for an effect of individual interventions, such as improved treatment of hypertension, dyslipidemia, and diabetes mellitus and other measures aimed at vascular factors.^25,43–45^ Multidomain interventions targeting multiple vascular risk factors increasingly are discussed as promising strategies for the prevention of degenerative dementias.^17,26^ It recently was estimated in a comprehensive review that the number of cases with Alzheimer's dementia could be reduced by about half by the elimination of the 7 most important modifiable risk factors.^2011^

Our results are compatible with the hypothesis of a dementia‐preventive effect of combined measures, but they can support it only indirectly. Several studies, such as the Dutch study Prevention of Dementia by Intensive Vascular Care (PreDIVA),^2009^ the French Multidomain Alzheimer Preventive Trial (MAPT),^2009^ and the Finnish Geriatric Intervention Study to Prevent Cognitive Impairment and Disability (FINGER) (http://www.ClinicalTrials.gov identifier NCT01041989), which likewise use a combination of lifestyle modifications and treatment of classical vascular risk factors as intervention, are under way and will be able to analyze the specific relationships to incident cognitive disorders.

Our study had several strengths. It was embedded in medical primary care, and it used methods that can be applied easily in general practice and are suitable for broad use at the population level. The generalizability of the results seems to be high, given that the members of a statutory health insurance program represent a sample of the general population and nearly all GPs practicing in the intervention district participated in the trial. The large sample size ensured high statistical power, which was additionally increased by the long duration of the trial. The clinical end point of LTC dependence, which is highly relevant for health policy, was determined independently of our trial by a medical service in the framework of German statutory LTC insurance.

Some limitations of our trial need to be mentioned. Relative contributions of preventive measures cannot be assessed reliably in multidomain interventions. Because of the aging of the population, LTC dependence gains importance and is an outstanding public health issue per se; nevertheless, although LTC dependence is caused mainly by stroke and dementia, it is a less specific end point than disease because it can develop as the result of many different diseases. It thus remains unclear to what extent prevention of stroke and dementia contributed to the reduction of LTC dependence in our study.

A cluster randomization of the GP practices in the study area over a long period of 8 years was not feasible because of insurants’ freedom to choose their physicians. We thus decided to compare the study group with insurants from a structurally similar district in Upper Bavaria. We were able to show that the morbidity rates in the 2 populations of insured did not differ initially. Neither in the year 2000, which preceded the trial, nor in the years of participant recruitment, from 2001 through 2004, were there significant differences in the incidence and prevalence of LTC dependence. Death rate differences in the 2 districts contributed just as little to the explanation of the positive effects in the evaluation period. Before the trial and during baseline, there were no significant differences in the death rates. It was possible to exclude death acting as a competing risk for the occurrence of LTC dependence. Rather, the results indicate that even the death rate in the intervention district was reduced after the intervention.

Data on vascular risk factors could be obtained only for those insured who enrolled in the study. For the nonparticipants and for the insured from the reference district who could not be examined in person, these data were not available. A comparison between the insured in the 2 districts at baseline and after the intervention period with regard to these data was thus not possible. The comparison between the regions is based exclusively on the outcome data available to the health insurer.

If different trends in lifestyle or in the occurrence of other vascular risk factors had arisen in the 2 districts during the trial, they could have contributed to the differences in the outcome. We are, however, not aware of any social, economic, or health policy changes that could have affected the districts differently.

In conclusion, the results of our trial indicate that a multidomain intervention, intended to prevent stroke and dementia, can reduce the incidence of LTC dependence in a real‐world setting. The public health impact of the 2 diseases possibly can be mitigated by improved control of vascular risk factors at the level of primary care. It will be an important task to investigate whether the effects are brought about primarily by the successful prevention of stroke or whether a multidomain intervention also contributes to the prevention of dementia.
